# Author Correction: Sparse inference and active learning of stochastic differential equations from data

**DOI:** 10.1038/s41598-024-57801-9

**Published:** 2024-03-29

**Authors:** Yunfei Huang, Youssef Mabrouk, Gerhard Gompper, Benedikt Sabass

**Affiliations:** 1 Theoretical Physics of Living Matter, Institute of Biological Information Processing and Institute for Advanced Simulation, Forschungszentrum Juelich, 52425 Juelich, Germany; 2 Department of Veterinary Sciences, Institute for Infectious Diseases and Zoonoses, Ludwig-Maximilians-Universitaet Munich, 80539 Munich, Germany; 3 Helmholtz Institute Muenster (HI MS), IEK-12 Forschungszentrum Juelich GmbH, Corrensstraße 46, 48149 Muenster, Germany

Correction to: *Scientific Reports* 10.1038/s41598-022-25638-9, published online 15 December 2022

The Data Availability section in the original version of this Article was incomplete. The original version of the statement,

“Source code and data can be obtained from the corresponding author upon request.”

now reads:

“Source code and data are available at 10.5281/zenodo.10732552.”

The original Article has been corrected.

